# Interpatient variability in the pharmacokinetics of remdesivir and its main metabolite GS-441524 in treated COVID-19 subjects

**DOI:** 10.1093/jac/dkac234

**Published:** 2022-07-15

**Authors:** Massimo Tempestilli, Tommaso Ascoli Bartoli, Domenico Benvenuto, Giulia Valeria Stazi, Luisa Marchioni, Emanuele Nicastri, Chiara Agrati

**Affiliations:** National Institute for Infectious Diseases ‘Lazzaro Spallanzani’ IRCCS, 00149, Rome, Italy; National Institute for Infectious Diseases ‘Lazzaro Spallanzani’ IRCCS, 00149, Rome, Italy; Infectious Disease Unit, Tor Vergata University Hospital, 00133, Rome, Italy; National Institute for Infectious Diseases ‘Lazzaro Spallanzani’ IRCCS, 00149, Rome, Italy; National Institute for Infectious Diseases ‘Lazzaro Spallanzani’ IRCCS, 00149, Rome, Italy; National Institute for Infectious Diseases ‘Lazzaro Spallanzani’ IRCCS, 00149, Rome, Italy; National Institute for Infectious Diseases ‘Lazzaro Spallanzani’ IRCCS, 00149, Rome, Italy

## Abstract

**Background:**

Remdesivir is the first antiviral drug against SARS-CoV-2 approved for use in COVID-19 patients.

**Objectives:**

To study the pharmacokinetic inter-individual variability of remdesivir and its main metabolite GS-441524 in a real-world setting of COVID-19 inpatients and to identify possible associations with different demographic/biochemical variables.

**Methods:**

Inpatients affected by SARS-CoV-2 infections, undergoing standard-dose remdesivir treatment, were prospectively enrolled. Blood samples were collected on day 4, immediately after (*C*_0_) and at 1 h (*C*_1_) and 24 h (*C*_24_) after infusion. Remdesivir and GS-441524 concentrations were measured using a validated UHPLC-MS/MS method and the AUC_0–24_ was calculated. At baseline, COVID-19 severity (ICU or no ICU), sex, age, BMI and renal and liver functions were assessed. Transaminases and estimated glomerular filtration rate (e-GFR) were also evaluated during treatment. Linear regression, logistic regression and multiple linear regression tests were used for statistical comparisons of pharmacokinetic parameters and variables.

**Results:**

Eighty-five patients were included. The mean (CV%) values of remdesivir were: *C*_0_ 2091 (99.1%) ng/mL, *C*_1_ 139.7 (272.4%) ng/mL and AUC_0–24_ 2791 (175.7%) ng·h/mL. The mean (CV%) values of GS-441524 were: *C*_0_ 90.2 (49.5%) ng/mL, *C*_1_ 104.9 (46.6%) ng/mL, *C*_24_ 58.4 (66.9) ng/mL and AUC_0–24_ 1976 (52.6%) ng·h/mL. The multiple regression analysis showed that age (*P < *0.05) and e-GFR (*P < *0.01) were independent predictors of GS-441524 plasma exposure.

**Conclusions:**

Our results showed a high interpatient variability of remdesivir and GS-441524 likely due to both age and renal function in COVID-19 inpatients. Further research is required to understand whether the pharmacokinetics of remdesivir and its metabolites may influence drug-related efficacy or toxic effect.

## Introduction

In early 2020, due to the urgent need to fight the coronavirus disease 2019 (COVID-19) pandemic, caused by severe acute respiratory syndrome coronavirus 2 (SARS-CoV-2), manufacturers rapidly initiated multiple studies on repurposing antiviral drugs as potential treatments.^1^ Among these, remdesivir is a prodrug of the nucleotide analogue GS-441524, with antiviral activity against several single-stranded RNA viruses, including Ebola, Marburg virus, HCV, MERS-CoV, SARS-CoV and SARS-CoV-2.^2^

Remdesivir has been shown to be possibly effective in improving COVID-19 outcomes in different settings, including inpatient and outpatient clinics.^3–6^ However, other studies did not confirm these encouraging findings and failed to provide conclusive data about remdesivir usefulness in COVID-19 clinical management.^7–10^

Potential pharmacokinetic (PK) inter-individual variability in the COVID-19 subjects may have contributed to different results obtained from clinical trials. Only few and small studies are currently available on remdesivir and metabolite PK in patients affected by SARS-CoV-2 infection.^11–15^

The aim of this research was to investigate the PK inter-individual variability of remdesivir and its adenine nucleoside analogue GS-441524 in inpatients affected by COVID-19. Furthermore, we evaluated the variables associated with prodrug and metabolite PK parameters, including liver injury (the most common adverse event observed among patients treated with remdesivir^16,17^).

## Methods

### Study design

This is a monocentric, prospective, observational study evaluating the PK of remdesivir and its main metabolite (GS-441524) in inpatients with moderate or severe COVID-19 infection. All patients were recruited at the National Institute for Infectious Diseases ‘Lazzaro Spallanzani’ between April and October 2020.

### Ethics

The study was conducted in accordance with the Declaration of Helsinki, as well as with national and institutional standards. Informed consent was obtained from family members. All data were collected anonymously.

### Patients and treatment

Patients aged ≥18 years, admitted to the acute infectious diseases unit or ICU, with microbiologically confirmed SARS-CoV-2 pneumonia, were included. IV remdesivir was administrated at a loading dose of 200 mg on day 1, followed by 100 mg daily from day 2 to 5 or 10, according to the evolving recommendations by the Italian drug agency. Serum ALT was measured at baseline, at day 4 after treatment start and at discharge. Estimated glomerular filtration rate (e-GFR) was calculated using the ‘Chronic Kidney Disease Epidemiology Collaboration’ (CKD-EPI) equation.

### Blood sampling and analytical method for PK assessments of remdesivir and GS-441524

For remdesivir and GS-441524 PK assessments at steady state, blood samples were collected on day 4, immediately after (*C*_0_) and at 1 h (*C*_1_) and 24 h (*C*_24_) after IV remdesivir administration.^18^

Plasma samples were obtained by centrifugation (5000 rpm for 5 min at +4°C) of blood samples (tubes containing lithium heparin). Immediately after collection, 1 mL of plasma was isolated and stored at –20°C until measurement of remdesivir and GS-441524 concentrations. Plasma concentrations of remdesivir and its metabolite GS-441524 were determined by using validated UHPLC-MS/MS.^19^ Briefly, 50 μL of sample was mixed with 600 μL of acetonitrile:methanol (50:50, v:v) containing internal standard (quinaxoline). Three hundred microlitres of supernatant was diluted with 600 μL of HPLC-MS grade water and 10 μL was injected into the column Acquity UPLC HSS T3 1.8 μm (2.1 × 50 mm Waters). Remdesivir and GS-441524 were measured simultaneously with a limit of quantification of 5.86 and 1.96 ng/mL, respectively. Linearity was 5.86 to 3000 ng/mL for remdesivir and 1.96 to 1000 ng/mL for GS-441524, with *R*^2^ >0.995 for both drugs. In order to decrease esterase plasma activity, all steps were performed on ice.

### Statistical comparisons of PK parameters and different categories of variables

Categorical variables are shown by frequency distributions and continuous variables by the mean and standard deviation or by the median and IQR. The AUC_0–24_ from the time of finish of infusion to the last infusion was calculated by Phoenix 8.1 using the Non Compartmental Analysis (linear log trapezoidal rule).

Statistically significant differences in terms of ALT and e-GFR values before and after the remdesivir infusion were evaluated using a Wilcoxon matched pairs test. Moreover, same statistical analysis was performed to evaluate the difference between ALT at baseline and at discharge.

Potential correlation between COVID-19 severity (ICU versus no ICU), sex, age, BMI, e-GFR, ALT and PK parameters of remdesivir and GS-441524 were evaluated using linear regression, logistic regression and multiple linear regression using the step-forward method. Statistical significance was set at *P* < 0.05 and for the regression only the *P* value related to the slope was considered and not the *P* value related to intercept because of little pharmacological interest.

Statistical analysis was performed using MedCalc Statistical Software version 19.2.6 (MedCalc Software bv, Ostend, Belgium; https://www.medcalc.org; 2020) and GraphPad Prism version 9.0.0 for Windows (GraphPad Software, San Diego, CA, USA; www.graphpad.com).

## Results

### Patient population

Eighty-five patients met the inclusion criteria and were enrolled in the study. The baseline characteristics of the patients are shown in Table 1. Previous treatments and drugs co-administered simultaneously with remdesivir were prescribed according to ongoing treatment guidelines.^20^ Based on drug metabolism and clearance a clinically significant drug–drug interaction was unlikely.^16,21^

**Table 1.** Baseline characteristics of the patients studied

Age (years), median (IQR)	59 (53–67)
Gender, *n* (%)	
male	58 (68.2)
female	27 (31.8)
BMI (kg/m^2^), median (IQR)	27.3 (25–30.8)
e-GFR (mL/min/1.73 m^2^), median (IQR)	93.6 (84.8–102.9)
ALT (IU), median (IQR)	32 (19.7–64.2)
Clinical status, *n* (%)	
severe (ICU)	54 (63.5)
moderate (no ICU)	31 (36.5)
Previous medications, *n* (%)	
hydroxychloroquine	9 (10.5)
lopinavir/ritonavir	8 (9.4)
tocilizumab	1 (1.2)
sirolimus	2 (2.4)
anakinra	1 (1.2)
Concomitant medications, *n* (%)	
corticosteroids	75 (88.2)
antiplatelet drug	6 (7.1)
low molecular weight heparin	85 (100)

Sixty-eight subjects (80%) received remdesivir for 5 days and 17 subjects (20%) received remdesivir for 10 days.

A significant increase between the ALT measured at baseline versus ALT at day 4 [42 IU (IQR 23–69)] was observed (*P < *0.01).

To understand the magnitude of the liver damage, patients’ ALT increase on day 4 from treatment initiation was evaluated. Three patients (3.5%) experienced a 5-fold rise of ALT during remdesivir treatment, while 7 (8.2%) and 27 (31.7%) individuals experienced a 2.5- to 5-fold and a 1.25- to 2.5-fold increase, respectively. In these patients, the liver enzyme returned to baseline values at hospital discharge (ALT at baseline versus ALT at discharge, *P* = 0.20).

In contrast, median e-GFR at day 4 [99.4 mL/min/1.73 m^2^ (IQR 89.3–104.5)] was improved with respect to baseline values (*P < *0.01).

Seventy-three patients (85.9%) experienced a full recovery and were subsequently discharged, while the remaining 12 (14.1%) patients died during their hospital stay.

### PK of remdesivir and main metabolite GS-441524 in COVID-19 patients

The remdesivir and GS-441524 PK parameters in COVID-19 inpatients (*n *= 85) are shown in Table 2 and in Figure S1 (available as Supplementary data at *JAC* Online).

**Table 2.** Mean (CV%) PK parameters of remdesivir and GS-441524 in 85 COVID-19 subjects

Parameter	Remdesivir	GS-441524
*C*_0_ (ng/mL)	2091 (99.1)	90.2 (49.5)
*C*_1_ (ng/mL)	139.7 (272.4)	104.9 (46.6)
*C*_24_ (ng/mL)	not detectable	58.4 (66.9)
AUC_0–24_ (ng·h/mL)	2791 (175.1)	1976 (52.6)

PK analysis showed that remdesivir concentration achieves its peak at the end of the IV infusion. Subsequently, the prodrug rapidly disappears from plasma, with a median half-life of <1 h. Conversely, the peak of GS-441524, the main remdesivir plasmatic metabolite, is reached about 1 h after the end of the infusion, with a prolonged half-life (>24 h).

### Variables influencing remdesivir and GS-441524 PK parameters

Possible association between biological variables (clinical status, sex, age, BMI, e-GFR, ALT) and AUC_0–24_ or single timepoints of remdesivir or GS-44124 was studied. Univariate analysis showed no significant associations between remdesivir and selected parameters. The results of the univariate analysis performed considering all the variables evaluated at every timepoint are reported in Table S1.

**Figure 1.**
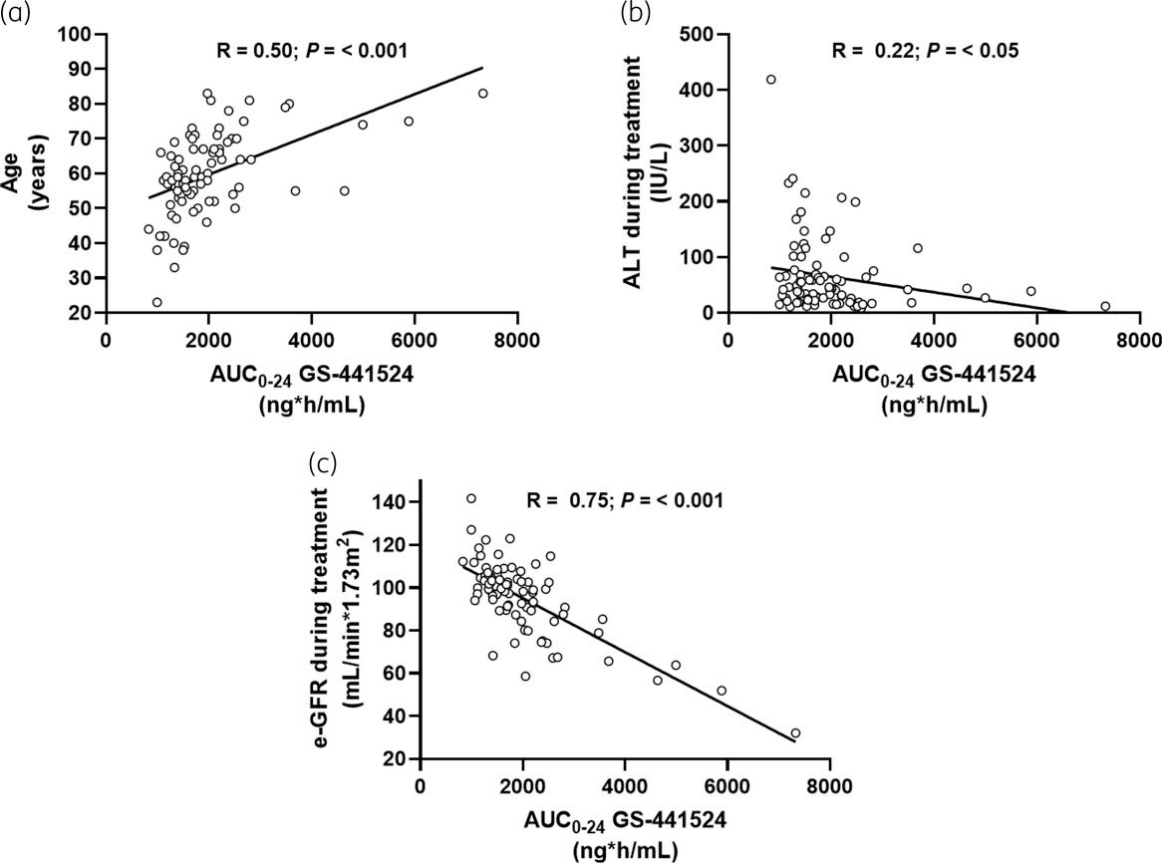
Linear regression curves and statistical results (univariate analysis) between GS-441524 plasma exposure and associated variables. (a) GS-441524 AUC_0–24_ and age. (b) GS-441524 AUC_0–24_ and ALT during remdesivir treatment. (c) GS-441524 AUC_0–24_ and e-GFR during remdesivir treatment. The whole linear regression analysis is reported in Table S1.

Linear regression analysis performed using GS-441524 AUC_0–24_ and age showed an *R*^2^ coefficient of 0.25, an *R* coefficient of 0.50 and *P < *0.001 (Figure 1a).

The same analysis, performed using GS-441524 AUC_0–24_ and ALT during the remdesivir treatment, showed an *R*^2^ coefficient of 0.05, an *R* coefficient of 0.22 and *P < *0.05 (Figure 1b).

Moreover, an inverse correlation between GS-441524 AUC_0–24_ and e-GFR at day 4 (*R*^2 ^= 0.56; *R *= 0.75; *P < *0.001) was found (Figure 1c).

In the multivariate linear regression analysis, age (coefficient 23.3; standard error 6.25; *P < *0.05) of the patients and e-GFR at day 4 (coefficient −38.7; standard error 11.38; *P < *0.01) were identified as independent predictors of GS-441524 plasma exposure, showing a coefficient of determination *R*^2^ of 0.57, a multiple correlation coefficient *R* of 0.76, an adjusted *R*^2^ of 0.56, a residual standard deviation of 735 and an F-ratio of the model of 30.63.

## Discussion

The current available PK evidence has been previously acquired in studies performed on healthy donors or from limited COVID-19 case series including few patients with peculiar characteristics.^11–15,22^ This is the largest data collection of time-dependent remdesivir and GS-441524 PK parameters in a real-life cohort of inpatients with COVID-19.

Our results confirmed remdesivir and GS-441524 PK characteristics (peak and half-life) previously observed in COVID-19 patients.^13,15,18^

In our study, the PK variability, measured following multiple doses of antiviral and expressed as CV%, was higher in COVID-19 patients than healthy donors. In particular, the CV% of systemic exposures (AUC_0–24_) in patients was about 10-fold (175.1% versus 19.1%) and 3-fold (52.6% versus 15.1%) greater than healthy donors for remdesivir and GS-441524, respectively.^22^

Metabolism and excretion are the most crucial and complex steps that can be the major source of PK variability, especially for IV drugs. Previous *in vivo* studies demonstrated that metabolism and glomerular filtration are the main routes of elimination for remdesivir and GS-441524, respectively.^16,21,22^

Some authors demonstrated that systemic inflammation can modify drug plasma metabolism.^23,24^ Therefore, the inflammatory profile related to SARS-CoV-2 infection could influence the remdesivir PK in COVID-19 patients.

The analysis reported here confirms that exposure to GS-441524 is increased in elderly patients or those with impaired renal function, supporting the hypothesis that the metabolite may not be adequately eliminated in the case of low e-GFR. This result is in line with previously published case reports.^11–15^

Renal function in COVID-19 subjects could be influenced by several factors, such as age, disease severity and drug treatments.^25–27^ Our population includes many patients presenting with severe or even critical conditions and with clinical characteristics that are more heterogeneous; it may at least partially explain the high interpatient metabolite PK variability observed in the cohort compared with the volunteers usually selected for PK studies.

The PK variability may have a potential effect in determining the efficacy of remdesivir administration in patients affected by COVID-19. According to this hypothesis, several authors suggested that the heterogeneous response to remdesivir could be caused by suboptimal lung exposure to the active metabolite, which appears to be strictly related to GS-441524 plasmatic concentration.^15,28^ Then, the suboptimal remdesivir antiviral effect observed in clinical trials may be explained by inadequate dosing; it’s still an open debate.^29,30^ Evaluation of the intracellular concentrations of the pharmacologically active metabolite triphosphate (GS-443902) in COVID-19 patients should be performed to demonstrate this theory.

Another interesting finding is related to drug-related toxicity. Although a reversible increase in ALT concentrations was observed during remdesivir treatment, the ALT values were all within the normal range in most cases. Liver injury marker elevation is the most frequently reported adverse effect in remdesivir clinical trials and an increase in ALT serum concentration is the most common.^16,17,31^

Multiple-ascending-dose Phase I studies indicate that remdesivir-associated transaminase elevation is dose dependent in healthy human volunteers.^22,29^ However, mild liver injury is also a common feature of COVID-19, even observed in patients not treated with remdesivir or other hepatotoxic drugs. Moreover, few studies reported a possible correlation between disease severity and rise of transaminases and other markers of liver damage.^32^ Our results did not identify any causal association between remdesivir or GS-441524 plasma exposure and rates of ALT increase in patients with moderate or severe COVID-19 pneumonia. Considering that the rise of transaminases was actually modest and of little clinical significance in our patients, it suggests that remdesivir-associated liver toxicity may have been previously overestimated and the liver injury is probably attributable to SARS-CoV-2 infection itself.^32^

The main limitations of our study were: (i) the lack of evaluation of association between PK and clinical or virological efficacy outcomes due to variability of the time of antiviral treatment initiation; (ii) the lack of intermediate timepoints between *C*_1_ and *C*_24_ and then potentially bias in calculated AUC_0–24_ values; and (iii) since this study was started during the first SARS-CoV-2 pandemic wave, the initial lack of knowledge regarding COVID-19 treatment should be considered.

In conclusion, our PK study added new information from a real-world setting on the variability of plasma exposure to remdesivir and its metabolite, identifying age and renal function as predictors of GS-441524 plasma concentration. Even further research is required in larger cohorts of COVID-19 patients to identify other variables that may influence the PK, clinical efficacy and toxicity of remdesivir and its metabolites; we strongly believe that this analysis could help to fill the gap in knowledge regarding the use of remdesivir to treat COVID-19 and will help to define therapeutic strategies more efficient and appropriate to treat SARS-CoV-2 infection.

## Supplementary Material

dkac234_Supplementary_Data

## Supplementary data

Figure S1 and Table S1 are available as Supplementary data at *JAC* Online.
