# Supplementary material for: Interpatient variability in the pharmacokinetics of remdesivir and its main metabolite GS-441524 in treated COVID-19 subjects
Source: J Antimicrob Chemother. 2022 Jul 15;77(10):2683–7. doi: 10.1093/jac/dkac234 (PMC9384469; doi:10.1093/jac/dkac234)
Supplement: dkac234_Supplementary_Data [file dkac234_supplementary_data.docx]

**Supplementary data**


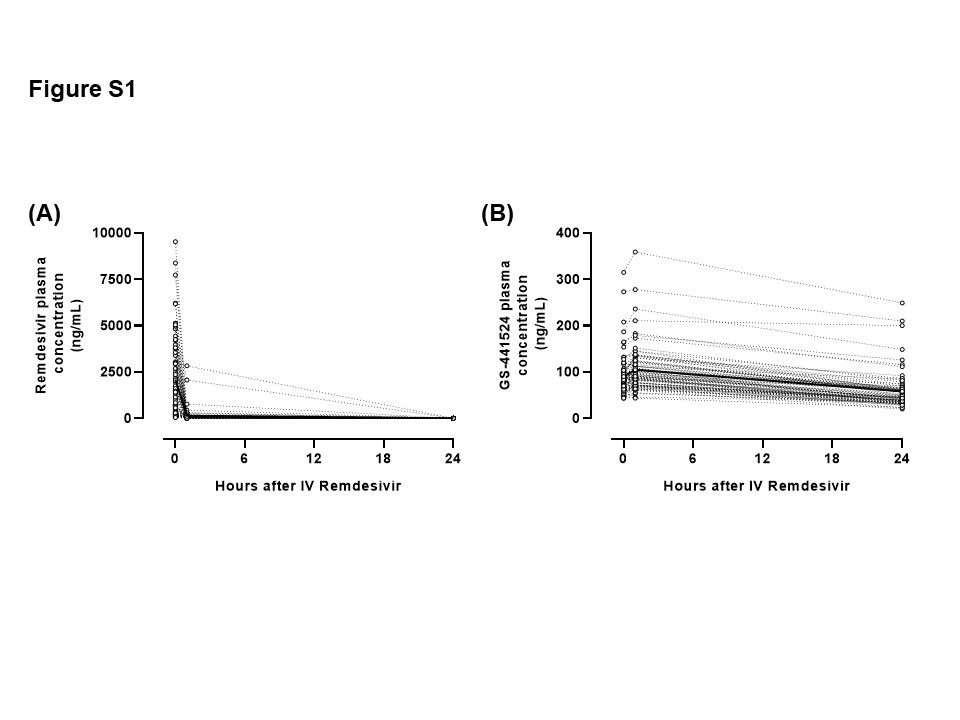


**Figure S1.** Plasma concentration-time profile and pharmacokinetic curves of remdesivir (A) and its metabolite GS-441524 (B) following antiviral intravenous administration on day 4. Individual concentration time points were measured immediately (C_0_) at 1 (C_1_), and 24 (C_24_) hours after drug infusion. Bold curves showed population mean.

| **Table S1. Univariate linear regression analysis** | | | | |
| --- | --- | --- | --- | --- |
|  | **Remdesivir pharmacokinetic parameters** | | | |
|  | **AUC_0-24_**  **ng•h/mL** | **C_0_**  **ng/mL** | **C_1_**  **ng/mL** | **C_24_**  **ng/mL** |
| **Sex (Male vs Female)** | 0,302 | 0,855 | 0,072 | na |
| **Age** | 0,350 | 0,095 | 0,494 | na |
| **ALT Baseline** | 0,805 | 0,544 | 0,675 | na |
| **e-GFR Baseline** | 0,705 | 0,460 | 0,758 | na |
| **ALT day 4** | 0,238 | 0,077 | 0,416 | na |
| **e-GFR day 4** | 0,614 | 0,462 | 0,692 | na |
| **Severity (ICU vs NO ICU)** | 0,386 | 0,376 | 0,568 | na |
| **BMI** | 0,981 | 0,741 | 0,952 | na |
|  |  | | | |
|  | **GS-441524 pharmacokinetic parameters** | | | |
|  | **AUC_0-24_**  **ng•h/mL** | **C_0_**  **ng/mL** | **C_1_**  **ng/mL** | **C_24_**  **ng/mL** |
| **Sex (Male vs Female)** | 0,1765 | 0,1219 | 0,1145 | 0,2978 |
| **Age** | **< 0.001** | **< 0.001** | **< 0.001** | **< 0.001** |
| **ALT Baseline** | 0,343 | 0,258 | 0,448 | 0,270 |
| **e-GFR Baseline** | **0,001** | **0,0015** | **0,003** | **0,001** |
| **ALT day 4** | **0,04** | **0,132** | **0,039** | **0,053** |
| **e-GFR day 4** | **< 0.001** | **< 0.001** | **< 0.001** | **< 0.001** |
| **Severity (ICU vs NO ICU)** | 0,169 | 0,206 | 0,126 | 0,279 |
| **BMI** | 0,454 | 0,507 | 0,540 | 0,391 |

The columns statistical analysis contain p-value from univariate analysis. Significant values were presented as bold text.
